# Ethyl (*Z*)-2-(2-fluoro­benzyl­idene)-7-methyl-3-oxo-5-phenyl-3,5-dihydro-2*H*-thia­zolo[3,2-*a*]pyrimidine-6-carboxyl­ate

**DOI:** 10.1107/S1600536811042899

**Published:** 2011-10-22

**Authors:** Cheng-Guang Zhao, Jie Hu, Ya-Li Zhang, Jing Zhang, Shu-Lin Yang

**Affiliations:** a Institute of Biotechnology, Nanjing University of Science and Technology, Nanjing, Jiangsu Province 210094, People’s Republic of China; b School of Pharmacy, Wenzhou Medical College, Wenzhou, Zhejiang Province 325035, People’s Republic of China

## Abstract

The title compound, C_23_H_19_FN_2_O_3_S, a fused-pyrimidine derivative, displays dihedral angles between the thia­zole ring and the benzene ring and substituted benzene ring of 7.10 (14) and 3.48 (12)°, respectively. The dihydro­pyrimidine ring adopts a flattened boat conformation. The olefinic double bond is in a *Z* configuration.

## Related literature

For related crystal structures, see: Hou (2009 ▶); Kulakov *et al.* (2009 ▶). For background to the biological properties of fused-pyrimidine derivatives, see: Alam *et al.* (2010 ▶); Al-Rashood & Abdel-Aziz (2010 ▶); Ashok *et al.* (2007 ▶); Jang *et al.* (2011 ▶); Wichmann *et al.* (1999 ▶);. Zhou *et al.* (2011 ▶).
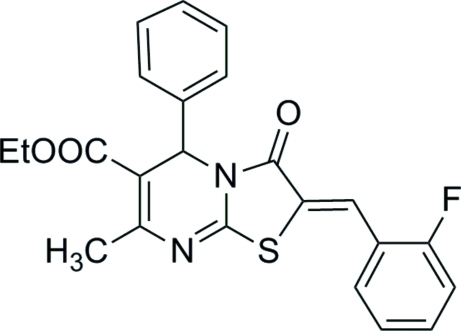


## Experimental

### 

#### Crystal data

C_23_H_19_FN_2_O_3_S
*M* *_r_* = 422.46
Monoclinic, 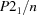

*a* = 9.3230 (19) Å
*b* = 10.170 (2) Å
*c* = 21.862 (4) Å
β = 96.33 (3)°
*V* = 2060.3 (7) Å^3^
*Z* = 4
Mo *K*α radiation
μ = 0.19 mm^−1^
*T* = 293 K
0.26 × 0.17 × 0.13 mm

#### Data collection

Bruker SMART diffractometer
Absorption correction: multi-scan (*SADABS*; Bruker, 2002 ▶) *T*_min_ = 0.831, *T*_max_ = 1.000
10963 measured reflections
4040 independent reflections
2918 reflections with *I* > 2σ(*I*)
*R*_int_ = 0.027

#### Refinement

*R*[*F* ^2^ > 2σ(*F* ^2^)] = 0.052
*wR*(*F* ^2^) = 0.147
*S* = 1.04
4040 reflections
273 parameters
H-atom parameters constrained
Δρ_max_ = 0.27 e Å^−3^
Δρ_min_ = −0.17 e Å^−3^

### 

Data collection: *SMART* (Bruker, 2002 ▶); cell refinement: *SAINT* (Bruker, 2002 ▶); data reduction: *SAINT*; program(s) used to solve structure: *SHELXS97* (Sheldrick, 2008 ▶); program(s) used to refine structure: *SHELXL97* (Sheldrick, 2008 ▶); molecular graphics: *SHELXTL* (Sheldrick, 2008 ▶); software used to prepare material for publication: *SHELXTL*.

## Supplementary Material

Crystal structure: contains datablock(s) I, global. DOI: 10.1107/S1600536811042899/ng5248sup1.cif

Structure factors: contains datablock(s) I. DOI: 10.1107/S1600536811042899/ng5248Isup2.hkl

Supplementary material file. DOI: 10.1107/S1600536811042899/ng5248Isup3.cml

Additional supplementary materials:  crystallographic information; 3D view; checkCIF report

## Figures and Tables

**Fig. 1.**
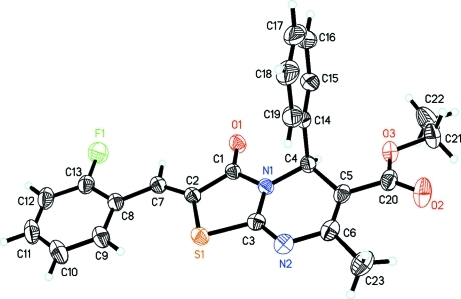
The molecular structure of the title compound, showing 30% displacement ellipsoids for the non-hydrogen atoms. Hydrogen atoms are drawn as spheres of arbitrary radius.

## supplementary crystallographic information

### Comment

Pyrimidine derivatives are important molecules owing to their useful biological and therapeutic activities (Ashok *et al.*, 2007; Zhou *et al.*, 2011). Thiazole derivatives have similar useful activity (Jang *et al.,* 2011). Such structural units are found in a vast number of naturally-occurring compounds and pharmaceuticals, so that the presence of both pyrimidine and thiazole rings give rise to enhanced activity (Al-Rashood & Abdel-Aziz, 2010; Wichmann *et al.,* 1999; Alam *et al.*, 2010).

In continuation of our studies on heterocyclic compounds, we report the crystal structure of (I). The fused thiazole ring has usual geometry as observed in other fused thiazolopyrimidine compounds (Hou, 2009; Kulakov *et al.*, 2009). The thiazole ring makes dihedral angles of 87.10 (14) and 3.48 (12) ° with the benzene rings C14–C17 and C8–C13, respectively. The pyrimidine ring adopts a flattened boat conformation. The C2—C7 double bond exist in the *Z* configuration. The crystal packing is stabilized by π-π stacking interactions. (Fig. 1).

### Experimental

In a one-pot Biginelli reaction, a mixture of 5 mmol of benzaldehyde, 6 mmol e thyl acetoacetate, 7.5 mmol thiourea and 10 ml of EtOH was stirred at 50°C in presence of sulfamic acid catalyst for 3 h to obtain 6-methyl-4-phenyl-2-thioxo-1,2,3,4- tetrahydropyrimidine-5-carboxylate. The product (2 mmol) was reacted with ethyl chloroacetate (2 mmol) in presence of pyridine for 4 h; 2-fluorobenzaldehyde (2 mmol) and piperidine were added, and and the mixture refluxed for 4 h until the TLC assay indicated that the reaction was completed. The reaction mixture was cooled and filtered to give the crude product. The solid was recystallized from acetic acid, and single crystals were grown in a CH_2_Cl_2_/CH_3_OH mixture (5:2 *v*/*v*)..

### Refinement

The H atoms were positioned geometrically (C—H = 0.93 and 0.96 Å) and refined as riding with *U*_ĩso_(H) = 1.2*U*_eq_(C) or 1.5*U*_eq_(methyl C).

### Crystal data

**Table:**

C_23_H_19_FN_2_O_3_S	*F*(000) = 880
*M**_r_* = 422.46	*D*_x_ = 1.362 Mg m^−^^3^
Monoclinic, *P*2_1_/*n*	Mo *K*α radiation, λ = 0.71073 Å
*a* = 9.3230 (19) Å	Cell parameters from 2127 reflections
*b* = 10.170 (2) Å	θ = 2.5–24.0°
*c* = 21.862 (4) Å	µ = 0.19 mm^−^^1^
β = 96.33 (3)°	*T* = 293 K
*V* = 2060.3 (7) Å^3^	Prismatic, green
*Z* = 4	0.26 × 0.17 × 0.13 mm

### Data collection

**Table:**

Bruker SMART diffractometer	4040 independent reflections
Radiation source: fine-focus sealed tube	2918 reflections with *I* > 2σ(*I*)
graphite	*R*_int_ = 0.027
ω scans	θ_max_ = 26.0°, θ_min_ = 1.9°
Absorption correction: multi-scan (*SADABS*; Bruker, 2002)	*h* = −11→11
*T*_min_ = 0.831, *T*_max_ = 1.000	*k* = −11→12
10963 measured reflections	*l* = −21→26

### Refinement

**Table:**

Refinement on *F*^2^	Primary atom site location: structure-invariant direct methods
Least-squares matrix: full	Secondary atom site location: difference Fourier map
*R*[*F*^2^ > 2σ(*F*^2^)] = 0.052	Hydrogen site location: inferred from neighbouring sites
*wR*(*F*^2^) = 0.147	H-atom parameters constrained
*S* = 1.04	*w* = 1/[σ^2^(*F*_o_^2^) + (0.0733*P*)^2^ + 0.4206*P*] where *P* = (*F*_o_^2^ + 2*F*_c_^2^)/3
4040 reflections	(Δ/σ)_max_ = 0.009
273 parameters	Δρ_max_ = 0.27 e Å^−^^3^
0 restraints	Δρ_min_ = −0.17 e Å^−^^3^

### Special details

**Table:**

Geometry. All e.s.d.'s (except the e.s.d. in the dihedral angle between two l.s. planes) are estimated using the full covariance matrix. The cell e.s.d.'s are taken into account individually in the estimation of e.s.d.'s in distances, angles and torsion angles; correlations between e.s.d.'s in cell parameters are only used when they are defined by crystal symmetry. An approximate (isotropic) treatment of cell e.s.d.'s is used for estimating e.s.d.'s involving l.s. planes.
Refinement. Refinement of *F*^2^ against ALL reflections. The weighted *R*-factor *wR* and goodness of fit *S* are based on *F*^2^, conventional *R*-factors *R* are based on *F*, with *F* set to zero for negative *F*^2^. The threshold expression of *F*^2^ > σ(*F*^2^) is used only for calculating *R*-factors(gt) *etc*. and is not relevant to the choice of reflections for refinement. *R*-factors based on *F*^2^ are statistically about twice as large as those based on *F*, and *R*- factors based on ALL data will be even larger.

### Fractional atomic coordinates and isotropic or equivalent isotropic displacement parameters (Å^2^)

**Table:**

	*x*	*y*	*z*	*U*_iso_*/*U*_eq_	
S1	0.12550 (6)	0.33812 (6)	1.02831 (3)	0.0515 (2)	
N1	0.00474 (18)	0.31076 (17)	0.91699 (8)	0.0421 (4)	
N2	−0.0764 (2)	0.16406 (19)	0.98954 (9)	0.0551 (5)	
F1	0.46561 (19)	0.74388 (17)	0.96836 (8)	0.0873 (5)	
O1	0.10315 (17)	0.47535 (17)	0.86461 (7)	0.0571 (4)	
O2	−0.3636 (2)	0.0068 (2)	0.83596 (11)	0.0977 (7)	
O3	−0.28690 (19)	0.1748 (2)	0.78413 (9)	0.0721 (5)	
C1	0.0939 (2)	0.4181 (2)	0.91203 (10)	0.0423 (5)	
C2	0.1745 (2)	0.4481 (2)	0.97289 (9)	0.0431 (5)	
C3	0.0027 (2)	0.2577 (2)	0.97443 (10)	0.0455 (5)	
C4	−0.0700 (2)	0.2454 (2)	0.86253 (10)	0.0476 (5)	
H4	−0.1203	0.3121	0.8358	0.057*	
C5	−0.1807 (2)	0.1507 (2)	0.88343 (11)	0.0499 (6)	
C6	−0.1772 (2)	0.1125 (2)	0.94227 (12)	0.0536 (6)	
C7	0.2703 (2)	0.5462 (2)	0.97837 (10)	0.0475 (5)	
H7	0.2781	0.5930	0.9424	0.057*	
C8	0.3638 (2)	0.5912 (2)	1.03149 (11)	0.0496 (6)	
C9	0.3658 (3)	0.5407 (3)	1.09065 (12)	0.0632 (7)	
H9	0.3031	0.4727	1.0979	0.076*	
C10	0.4586 (3)	0.5889 (3)	1.13895 (13)	0.0768 (9)	
H10	0.4578	0.5534	1.1781	0.092*	
C11	0.5515 (3)	0.6883 (3)	1.12944 (16)	0.0795 (9)	
H11	0.6140	0.7202	1.1622	0.095*	
C12	0.5536 (3)	0.7414 (3)	1.07228 (15)	0.0747 (8)	
H12	0.6164	0.8096	1.0657	0.090*	
C13	0.4611 (3)	0.6919 (3)	1.02485 (13)	0.0588 (6)	
C14	0.0385 (3)	0.1753 (3)	0.82699 (11)	0.0574 (7)	
C15	0.0600 (3)	0.2140 (3)	0.76873 (13)	0.0809 (9)	
H15	0.0088	0.2847	0.7504	0.097*	
C16	0.1604 (5)	0.1456 (5)	0.73671 (18)	0.1075 (14)	
H16	0.1754	0.1707	0.6970	0.129*	
C17	0.2346 (4)	0.0434 (5)	0.7641 (2)	0.1163 (17)	
H17	0.3004	−0.0013	0.7428	0.140*	
C18	0.2155 (3)	0.0046 (4)	0.8219 (2)	0.1027 (13)	
H18	0.2684	−0.0653	0.8402	0.123*	
C19	0.1160 (3)	0.0704 (3)	0.85343 (15)	0.0768 (9)	
H19	0.1014	0.0434	0.8929	0.092*	
C20	−0.2869 (3)	0.1001 (3)	0.83382 (14)	0.0605 (7)	
C21	−0.3860 (3)	0.1400 (4)	0.73092 (15)	0.0931 (11)	
H21A	−0.3472	0.0674	0.7092	0.112*	
H21B	−0.4777	0.1128	0.7439	0.112*	
C22	−0.4067 (5)	0.2525 (5)	0.69102 (16)	0.1257 (15)	
H22A	−0.4401	0.3254	0.7135	0.189*	
H22B	−0.4769	0.2320	0.6569	0.189*	
H22C	−0.3169	0.2751	0.6761	0.189*	
C23	−0.2756 (3)	0.0149 (3)	0.96687 (15)	0.0743 (8)	
H23A	−0.3639	0.0100	0.9399	0.111*	
H23B	−0.2961	0.0418	1.0071	0.111*	
H23C	−0.2300	−0.0699	0.9695	0.111*	

### Atomic displacement parameters (Å^2^)

**Table:**

	*U*^11^	*U*^22^	*U*^33^	*U*^12^	*U*^13^	*U*^23^
S1	0.0556 (4)	0.0553 (4)	0.0424 (3)	0.0045 (3)	−0.0004 (3)	0.0014 (3)
N1	0.0425 (10)	0.0440 (10)	0.0396 (9)	0.0018 (8)	0.0034 (7)	−0.0048 (8)
N2	0.0538 (12)	0.0497 (12)	0.0619 (13)	0.0032 (10)	0.0064 (10)	0.0065 (10)
F1	0.0901 (12)	0.0884 (12)	0.0848 (12)	−0.0292 (10)	0.0158 (9)	−0.0101 (9)
O1	0.0642 (10)	0.0621 (10)	0.0435 (9)	−0.0110 (8)	−0.0008 (7)	0.0053 (8)
O2	0.0845 (15)	0.0827 (15)	0.1215 (19)	−0.0357 (12)	−0.0082 (13)	−0.0088 (13)
O3	0.0576 (11)	0.0893 (14)	0.0668 (12)	−0.0195 (10)	−0.0052 (9)	−0.0159 (10)
C1	0.0424 (12)	0.0417 (12)	0.0423 (12)	0.0036 (9)	0.0027 (9)	−0.0044 (10)
C2	0.0434 (12)	0.0439 (12)	0.0413 (12)	0.0118 (10)	0.0023 (9)	−0.0061 (9)
C3	0.0439 (12)	0.0443 (12)	0.0487 (13)	0.0104 (10)	0.0063 (10)	0.0013 (10)
C4	0.0418 (12)	0.0527 (13)	0.0473 (12)	−0.0001 (10)	0.0004 (10)	−0.0083 (10)
C5	0.0360 (12)	0.0456 (12)	0.0684 (16)	0.0033 (10)	0.0076 (11)	−0.0087 (11)
C6	0.0433 (13)	0.0409 (12)	0.0772 (18)	0.0069 (10)	0.0091 (12)	0.0008 (12)
C7	0.0472 (12)	0.0469 (12)	0.0475 (12)	0.0075 (10)	0.0009 (10)	−0.0060 (10)
C8	0.0432 (12)	0.0501 (13)	0.0542 (14)	0.0116 (10)	−0.0009 (10)	−0.0119 (11)
C9	0.0656 (16)	0.0600 (16)	0.0605 (16)	0.0053 (13)	−0.0085 (12)	−0.0130 (12)
C10	0.081 (2)	0.082 (2)	0.0614 (17)	0.0164 (17)	−0.0211 (15)	−0.0173 (15)
C11	0.0549 (17)	0.088 (2)	0.090 (2)	0.0123 (16)	−0.0197 (15)	−0.0418 (18)
C12	0.0486 (15)	0.0779 (19)	0.095 (2)	0.0000 (14)	−0.0020 (15)	−0.0379 (17)
C13	0.0459 (13)	0.0580 (15)	0.0719 (17)	0.0036 (12)	0.0041 (12)	−0.0179 (13)
C14	0.0435 (13)	0.0694 (16)	0.0598 (15)	−0.0153 (12)	0.0084 (11)	−0.0282 (13)
C15	0.080 (2)	0.098 (2)	0.0684 (18)	−0.0322 (17)	0.0249 (15)	−0.0315 (16)
C16	0.106 (3)	0.132 (3)	0.094 (3)	−0.059 (3)	0.053 (2)	−0.055 (3)
C17	0.075 (2)	0.128 (4)	0.155 (4)	−0.037 (2)	0.056 (3)	−0.087 (3)
C18	0.0606 (19)	0.109 (3)	0.140 (3)	0.0046 (18)	0.020 (2)	−0.057 (3)
C19	0.0515 (15)	0.085 (2)	0.094 (2)	0.0080 (15)	0.0098 (14)	−0.0357 (17)
C20	0.0411 (13)	0.0565 (15)	0.0834 (19)	−0.0013 (12)	0.0050 (12)	−0.0158 (14)
C21	0.071 (2)	0.121 (3)	0.081 (2)	−0.0205 (19)	−0.0181 (17)	−0.026 (2)
C22	0.160 (4)	0.138 (4)	0.069 (2)	−0.015 (3)	−0.030 (2)	−0.018 (2)
C23	0.0620 (17)	0.0546 (16)	0.107 (2)	−0.0019 (13)	0.0147 (15)	0.0184 (15)

### Geometric parameters (Å, °)

**Table:**

S1—C2	1.746 (2)	C10—C11	1.361 (4)
S1—C3	1.752 (2)	C10—H10	0.9300
N1—C3	1.369 (3)	C11—C12	1.364 (4)
N1—C1	1.384 (3)	C11—H11	0.9300
N1—C4	1.470 (3)	C12—C13	1.369 (4)
N2—C3	1.270 (3)	C12—H12	0.9300
N2—C6	1.418 (3)	C14—C15	1.368 (4)
F1—C13	1.348 (3)	C14—C19	1.379 (4)
O1—C1	1.201 (3)	C15—C16	1.413 (5)
O2—C20	1.192 (3)	C15—H15	0.9300
O3—C20	1.326 (3)	C16—C17	1.351 (6)
O3—C21	1.448 (3)	C16—H16	0.9300
C1—C2	1.486 (3)	C17—C18	1.353 (6)
C2—C7	1.336 (3)	C17—H17	0.9300
C4—C5	1.518 (3)	C18—C19	1.387 (4)
C4—C14	1.520 (3)	C18—H18	0.9300
C4—H4	0.9800	C19—H19	0.9300
C5—C6	1.341 (3)	C21—C22	1.438 (5)
C5—C20	1.478 (3)	C21—H21A	0.9700
C6—C23	1.492 (3)	C21—H21B	0.9700
C7—C8	1.447 (3)	C22—H22A	0.9600
C7—H7	0.9300	C22—H22B	0.9600
C8—C13	1.387 (4)	C22—H22C	0.9600
C8—C9	1.390 (4)	C23—H23A	0.9600
C9—C10	1.379 (4)	C23—H23B	0.9600
C9—H9	0.9300	C23—H23C	0.9600
			
C2—S1—C3	91.96 (11)	C13—C12—H12	120.8
C3—N1—C1	116.89 (18)	F1—C13—C12	118.0 (3)
C3—N1—C4	120.74 (18)	F1—C13—C8	118.2 (2)
C1—N1—C4	121.90 (18)	C12—C13—C8	123.8 (3)
C3—N2—C6	116.4 (2)	C15—C14—C19	119.3 (3)
C20—O3—C21	117.4 (2)	C15—C14—C4	121.0 (3)
O1—C1—N1	123.46 (19)	C19—C14—C4	119.7 (2)
O1—C1—C2	126.6 (2)	C14—C15—C16	119.5 (4)
N1—C1—C2	109.93 (19)	C14—C15—H15	120.2
C7—C2—C1	120.1 (2)	C16—C15—H15	120.2
C7—C2—S1	129.77 (17)	C17—C16—C15	119.6 (4)
C1—C2—S1	110.16 (16)	C17—C16—H16	120.2
N2—C3—N1	127.0 (2)	C15—C16—H16	120.2
N2—C3—S1	122.04 (18)	C16—C17—C18	121.7 (4)
N1—C3—S1	111.00 (16)	C16—C17—H17	119.2
N1—C4—C5	108.68 (18)	C18—C17—H17	119.2
N1—C4—C14	110.08 (17)	C17—C18—C19	119.1 (4)
C5—C4—C14	111.69 (19)	C17—C18—H18	120.4
N1—C4—H4	108.8	C19—C18—H18	120.4
C5—C4—H4	108.8	C14—C19—C18	120.9 (4)
C14—C4—H4	108.8	C14—C19—H19	119.6
C6—C5—C20	123.1 (2)	C18—C19—H19	119.6
C6—C5—C4	121.8 (2)	O2—C20—O3	122.9 (3)
C20—C5—C4	115.0 (2)	O2—C20—C5	127.2 (3)
C5—C6—N2	122.4 (2)	O3—C20—C5	109.9 (2)
C5—C6—C23	126.0 (2)	C22—C21—O3	108.9 (3)
N2—C6—C23	111.6 (2)	C22—C21—H21A	109.9
C2—C7—C8	130.2 (2)	O3—C21—H21A	109.9
C2—C7—H7	114.9	C22—C21—H21B	109.9
C8—C7—H7	114.9	O3—C21—H21B	109.9
C13—C8—C9	115.4 (2)	H21A—C21—H21B	108.3
C13—C8—C7	119.5 (2)	C21—C22—H22A	109.5
C9—C8—C7	125.1 (2)	C21—C22—H22B	109.5
C10—C9—C8	121.6 (3)	H22A—C22—H22B	109.5
C10—C9—H9	119.2	C21—C22—H22C	109.5
C8—C9—H9	119.2	H22A—C22—H22C	109.5
C11—C10—C9	120.2 (3)	H22B—C22—H22C	109.5
C11—C10—H10	119.9	C6—C23—H23A	109.5
C9—C10—H10	119.9	C6—C23—H23B	109.5
C10—C11—C12	120.5 (3)	H23A—C23—H23B	109.5
C10—C11—H11	119.7	C6—C23—H23C	109.5
C12—C11—H11	119.7	H23A—C23—H23C	109.5
C11—C12—C13	118.5 (3)	H23B—C23—H23C	109.5
C11—C12—H12	120.8		
			
C3—N1—C1—O1	−178.3 (2)	S1—C2—C7—C8	0.6 (4)
C4—N1—C1—O1	9.5 (3)	C2—C7—C8—C13	176.4 (2)
C3—N1—C1—C2	2.4 (2)	C2—C7—C8—C9	−3.3 (4)
C4—N1—C1—C2	−169.82 (17)	C13—C8—C9—C10	0.2 (4)
O1—C1—C2—C7	−1.2 (3)	C7—C8—C9—C10	179.9 (2)
N1—C1—C2—C7	178.14 (18)	C8—C9—C10—C11	−0.1 (4)
O1—C1—C2—S1	−179.97 (19)	C9—C10—C11—C12	0.2 (4)
N1—C1—C2—S1	−0.6 (2)	C10—C11—C12—C13	−0.4 (4)
C3—S1—C2—C7	−179.4 (2)	C11—C12—C13—F1	−179.1 (2)
C3—S1—C2—C1	−0.79 (16)	C11—C12—C13—C8	0.6 (4)
C6—N2—C3—N1	−2.6 (3)	C9—C8—C13—F1	179.2 (2)
C6—N2—C3—S1	176.41 (16)	C7—C8—C13—F1	−0.5 (3)
C1—N1—C3—N2	176.1 (2)	C9—C8—C13—C12	−0.5 (4)
C4—N1—C3—N2	−11.6 (3)	C7—C8—C13—C12	179.8 (2)
C1—N1—C3—S1	−3.0 (2)	N1—C4—C14—C15	−115.2 (2)
C4—N1—C3—S1	169.30 (15)	C5—C4—C14—C15	124.0 (2)
C2—S1—C3—N2	−177.05 (19)	N1—C4—C14—C19	65.4 (3)
C2—S1—C3—N1	2.07 (16)	C5—C4—C14—C19	−55.4 (3)
C3—N1—C4—C5	19.3 (3)	C19—C14—C15—C16	0.2 (4)
C1—N1—C4—C5	−168.82 (18)	C4—C14—C15—C16	−179.2 (2)
C3—N1—C4—C14	−103.3 (2)	C14—C15—C16—C17	−0.4 (5)
C1—N1—C4—C14	68.6 (3)	C15—C16—C17—C18	−0.1 (6)
N1—C4—C5—C6	−16.0 (3)	C16—C17—C18—C19	0.8 (5)
C14—C4—C5—C6	105.6 (2)	C15—C14—C19—C18	0.4 (4)
N1—C4—C5—C20	166.70 (18)	C4—C14—C19—C18	179.8 (2)
C14—C4—C5—C20	−71.7 (2)	C17—C18—C19—C14	−0.9 (5)
C20—C5—C6—N2	−178.7 (2)	C21—O3—C20—O2	0.4 (4)
C4—C5—C6—N2	4.3 (3)	C21—O3—C20—C5	−179.5 (2)
C20—C5—C6—C23	0.5 (4)	C6—C5—C20—O2	−13.9 (4)
C4—C5—C6—C23	−176.5 (2)	C4—C5—C20—O2	163.4 (3)
C3—N2—C6—C5	6.2 (3)	C6—C5—C20—O3	166.0 (2)
C3—N2—C6—C23	−173.1 (2)	C4—C5—C20—O3	−16.8 (3)
C1—C2—C7—C8	−177.9 (2)	C20—O3—C21—C22	160.5 (3)

### Hydrogen-bond geometry (Å, °)

**Table:**

*D*—H···*A*	*D*—H	H···*A*	*D*···*A*	*D*—H···*A*
C17—H17···O1^i^	0.93	2.62	3.410 (5)	144.
C23—H23C···S1^ii^	0.96	2.90	3.851 (3)	173.

Symmetry codes: (i) −*x*+1/2, *y*−1/2, −*z*+3/2; (ii) −*x*, −*y*, −*z*+2.
